# Long non-coding RNA ZNF674-AS1 antagonizes oxaliplatin resistance of gastric cancer via regulating EZH2-mediated methylation of CHST7

**DOI:** 10.18632/aging.204165

**Published:** 2022-07-08

**Authors:** Kun Ye, Yong Wang

**Affiliations:** 1 Department of General Surgery, The Second Affiliated Hospital of Anhui Medical University, Hefei 230022, Anhui Province, China

**Keywords:** ZNF674-AS1, CHST7, EZH2, gastric cancer, oxaliplatin resistance

## Abstract

Chemoresistance leads the cause of poor outcome of patients with gastric cancer (GC). Long non-coding RNAs (LncRNAs) is intimately involved in the regulation of tumorigenesis and progression. Here, we demonstrated ZNF674-AS1 was down-regulated in oxaliplatin (OXA)-resistant tissues and cell lines, lower level of ZNF674-AS1 predicted poor prognosis of GC patients. Besides, forced expression of ZNF674-AS1 not only reduced cell viability, colony formation, expression of drug-resistant markers but also promoted cell apoptosis of OXA-resistant GC cells, exposed to oxaliplatin. Silence of ZNF674-AS1 exhibited an opposite effects on OXA resistance of GC cells. Further mechanistic research showed that ZNF674-AS1 interacted with EZH2, led to higher methylation level of target gene CHST7. In addition, functional experiments verified that depletion of CHST7 re-sensitized OXA-resistant GC cells to OXA. Thus, our results indicated that ZNF674-AS1 suppressed OXA resistance of GC through EZH2-mediated inhibition of CHST7, providing potential theoretic basis and therapeutic strategy for chemoresistant GC.

## INTRODUCTION

Gastric cancer (GC) seriously threatens people’s life for the very unsatisfactory prognosis globally [1, 2]. Compared to targeted therapy and surgery, chemotherapy is still the preferred routine treatment strategy for advanced GC patients to reduce postoperative recurrence and prolong survival [3]. For example, oxaliplatin (OXA) is a common agent for the therapy of multiple cancers, GC is no exception [4, 5]. Nevertheless, the occurrence of OXA resistance greatly reduces the therapeutic efficiency of chemotherapy [6]. Therefore, the molecular mechanisms of OXA resistance in GC need to be urgently explored.

LncRNAs, a kind of >200 bp RNA transcripts with less protein-coding capacity, [7] mediate multiple critical cellular processes including proliferation, metastasis, and drug resistance in tumor aggressiveness [8, 9]. For example, up-regulation of LncRNA NEAT1 induces taxol and cisplatin resistance of breast cancer [10]. MiR-363/ABCC1 axis mediates LncRNA NR2F1-AS1-induced OXA resistance in hepatocellular carcinoma [11].

EZH2, one core member of the Polycomb Repressive Complex 2 (PRC2), acts as a critical regulator in epigenetic mediation of genes via catalyzing trimethylation of Lys-27 of histone H3 (H3K27me3), which serves as a crucial mediator in cancer development and aggressiveness. Elevated EZH2 has been observed in multiple solid tumor types, GC included; high level of EZH2 predicts poor outcome of cancer patients [12, 13]. Increasing studies indicate LncRNAs participate in EZH2-mediated H3K27me3, resulting in inhibition of target gene epigenetically [14, 15]. Here, we concerned a less-studied LncRNA, ZNF674-AS1, which was reported in hepatocellular, non-small cell lung cancer [16] and glioma cancers [17, 18]. However, the specific functional mechanisms of ZNF674-AS1 in GC are still vague.

In the present work, we show the tumor-suppressive phenotypes of ZNF674-AS1 in GC, inhibition of which enhanced OVA resistance of GC cells. Mechanism studies indicated the inhibitory effects of ZNF674-AS1 on OVA resistance depended on EZH2-induced epigenetically inhibition of CHST7 expression.

## MATERIALS AND METHODS

### Clinical tissues

70 GC tumor sample tissues (31 from OVA-resistant GC patients and 39 from OVA-sensitive GC patients) were collected from the Department of General Surgery, the Second Affiliated Hospital of Anhui Medical University. Informed consent has been signed by each enrolled patient before surgery. Sample collection and usage were performed according to the relevant guidelines.

### Cell culture and treatment

All cell lines used were cultured as recommended [19]. OXA-resistant GC cells (AGS/OXA, MKN-45/OXA, and MKN-28/OXA) were constructed according to the previous reports [20].

### Manipulation of gene expression

The full-length ZNF674-AS1 was amplified and inserted into pSin vector as recommended [21, 22]. shRNAs against ZNF674-AS1 were designed and inserted into pLKO.1 plasmid. ZNF674-AS1 overexpression or knockdown GC cells were constructed by infection of lentivirus expressing ZNF674-AS1 (pSin-ZNF674-AS1) or shRNAs against ZNF674-AS1, puromycin was used to select stable cell lines. Lipofectamine 3000 (Thermo Fisher Scientific) was used for transfection of siRNAs against CHST7 and EZH2 as recommended:

sh-ZNF674-AS1-1-F: ccggGACCATTTCCAACGGTTAACTggatccAGTTAACCGTTGGAAATGGTCtttttg

sh-ZNF674-AS1-1-R: aattcaaaaaGACCATTTCCAACGGTTAACTggatccAGTTAACCGTTGGAAATGGTC

sh-ZNF674-AS1-2-F: ccggGTTTAGAGATACCAACCTATTggatccAATAGGTTGGTATCTCTAAACtttttg

sh-ZNF674-AS1-2-R: aattcaaaaaGTTTAGAGATACCAACCTATTggatccAATAGGTTGGTATCTCTAAAC

si-CHST7-1: GGCGGACTAACAAGGTCATCT.

si-CHST7-2: CCCGGGGGCCAGTCTCGCGCG.

### Quantitative real-time PCR

Trizol reagent (Takara, Dalian, China) was used for extraction of total RNA from GC tissue specimens and cells as the recommendation of manufacturer’s manual. qRT-PCR was carried out as previously described [23]. The primers were listed:

ZNF674-AS1 F: 5′-CAAAGCCTGTGGCCGATGTG-3′;

ZNF674-AS1 R: 5′-ATGGTCACACATTCCTTCTCCC-3′;

CHST7 F: 5′-TTCAACCAGCACCCGGACG-3′;

CHST7 R: 5′-GCACAGAGAAATCACAGCGGAAG-3′;

CHST7 promoter F: 5′-CAGTGAGTGGTGATAGTGCCACCGC-3′;

CHST7 promoter F: 5′-TAATCTACAACTTTCCCTT-3′;

MDR1 F: 5′-GTTGCCATTGACTGAA AGAAC-3′;

MDR1 R: 5′-ACAGGAGATAGGCTGGTTTGA-3′;

MRP1 F: 5′-CTCCCCGGTCTATTCCCATTTCAA-3′;

MRP1 R: 5′-TCTCGGTAGCGCAGGCAGTAGTTC-3′;

LRP1 F: 5′-TCTACTTTGCCG-ACACCACC-3′;

LRP1 R: 5′-TGTCTTTTTGGGCCCATCGT-3′.

### Western blotting

RIPA buffer (Beyotime, Shanghai, China) pretreated with protease inhibitors (Beyotime, Shanghai, China) was used to extract total proteins from GC cells. Western blotting was conducted as previously described [23]. Primary antibodies against β-actin, EZH2 and CHST7 were purchased from Abcam (Cambridge, United Kingdom).

### Cell function assays

Cell proliferation of GC cells was detected with CCK-8 kit (Solarbio, Beijing, China) following the instruction. The apoptosis of GC cells was performed by using the Caspase-3 Colorimetric Assay kit from Promega Corporation. Colony formation assay was conducted as previously described [24].

### Isolation of nuclear and cytoplasmic RNA

RNA cellular sublocalization was analyzed using a Purification Kit (NorgenBiotek, Canada) according to the manuals of manufacturers.

### RNA pull-down assay

The detailed protocol of RNA pull-down assay was described previously [25]. Probes against ZNF674-AS1 were listed as below:

ZNF674-AS1-DNA-1-sense:

(biotin-) GGGTTAA TATGTCCTGA GGAGAGTAAA GAAA,

ZNF674-AS1-DNA-1-antisense:

(biotin-) TTTCTTTACTCTCCTCAGGACATATTAACCC;

ZNF674-AS1-DNA-2-sense:

(biotin-) ACTTCACCAT AATACCGGTT AATGTGATAA,

ZNF674-AS1-DNA-2-antisense:

(biotin-) TTATCACATTAACCGGTATTATGGTGAAGT;

ZNF674-AS1-DNA-3-sense:

(biotin-) TCAAAATCT CTATTCTTGA GCACAGTAGT,

ZNF674-AS1-DNA-3-antisense:

(biotin-) ACTACTGTGCTCAAGAATAGAGATTTTGA.

### RNA immunoprecipitation (RIP) and chromatin immunoprecipitation (ChIP)

RIP and ChIP assay were performed as previously described [26]. Antibodies targeting EZH2 and control IgG were purchased from Abcam.

### Bisulfite sequencing PCR (BSP)

Methylation level of CHST7 promoter was examined as recommended previously [26]. Primers used were listed: methylated CHST7 F: 5′- TTTGAAGTTATTGATGAAGATTTTATTAC-3′, R: 5′- CCTATTAACCGACTTAACAACCG-3′; unmethylated CHST7 F: 5′- GTTTGAAGTTATTGATGAAGATTTTATTAT-3′, R: 5′- CCTATTAACCAACTTAACAACCACC-3′.

### Statistical analysis

All data are presented as mean ± standard deviation (SD). Chi-squared test was used for analysis of association between ZNF674-AS1 expression and clinicopathological characteristics of GC patients. Student’s t-test and two-way ANOVA were used for statistical analysis between groups. Kaplan-Meier’s analysis was employed for statistical comparison of survival rate. P < 0.05 was considered to be statistically significant.

### Ethics approval and consent to participate

The study was approved by the ethics committee of the Second Affiliated Hospital of Anhui Medical University, Anhui Medical University.

### Data statement

Data will be provided on reasonable request.

## RESULTS

### Reduced ZNF674-AS1 correlated to oxaliplatin resistance and worse survival in gastric cancer

**Figure 1:**
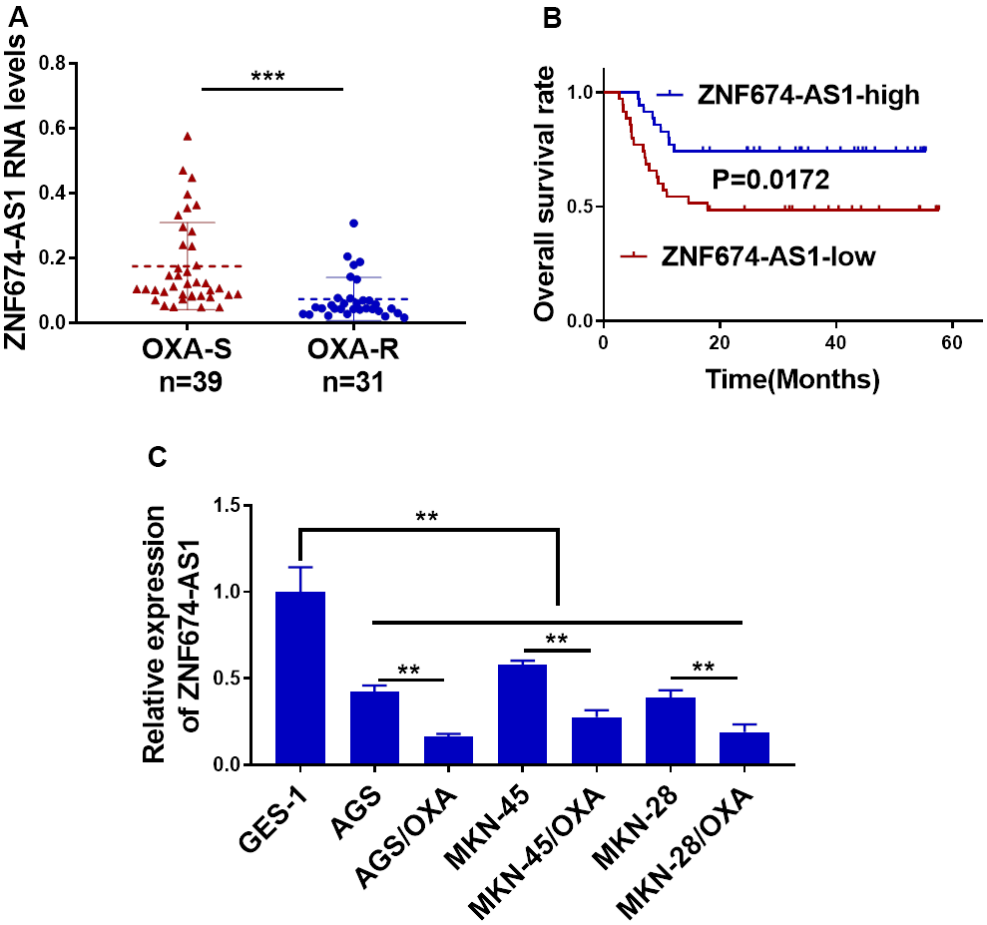
**Reduced ZNF674-AS1 correlated to oxaliplatin resistance and poor outcome in gastric cancer.** (**A**) RNA level of ZNF674-AS1 in 31 oxaliplatin-resistant GC tissues and 39 oxaliplatin-sensitive GC tissues was examined by qRT-PCR. (**B**) Overall survival of GC patients with lower or higher level of ZNF674-AS1was determined by Kaplan-Meier’s analysis. (**C**) ZNF674-AS1 expression in six GC cell lines (oxaliplatin-resistant cells or parental AGS, MKN-45, and MKN-28) and normal human gastric epithelial cell lines (GES) was determined by qRT-PCR. Student’s t-test for (**A**, **C**).

To explore the potentially dysregulation of ZNF674-AS1 in OVA-resistant gastric cancer, we first assessed ZNF674-AS1 expression in OVA-resistant GC samples and the OVA-sensitive counterparts by qRT-PCR. A significantly lower ZNF674-AS1 level was observed in OXA-resistant samples than that in OXA-sensitive ones (Figure 1A). We analyzed the survival rate of 70 GC patients, which indicated patient with a lower ZNF674-AS1 expression exhibited a worse outcome (Figure 1B). Further, pathological examination in Table 1 indicated elevated ZNF674-AS1 in gastric cancer was negatively associated with advanced WHO stage (P=0.0312) and lymphovascular invasion (0.0147). In keeping with the results of GC tissues, ZNF674-AS1 was remarkably down-regulated in OVA-resistant GC cells, as compared with their parental ones (Figure 1C). Collectively, these data suggested expression inhibition of ZNF674-AS1 in GC may be mechanistically correlated to OXA resistance and worse clinical outcomes.

**Table 1:** Association between ZNF674-AS1 expression and clinical characteristic parameters of 70 gastric cancer patients.

**Expression of ZNF674-AS1**			
**Variables**	**Low (%)**	**High (%)**	**P value**
Age			0.4452
<60	13(56.52%)	10(43.48%)	
≥60	22(46.81%)	25(53.19%)	
Gender			0.2195
Male	24(55.81%)	19(44.19%)	
Female	11(40.74%)	16(59.26%)	
TNM staging			0.0312*
I-II	14(37.84%)	23(62.16%)	
III-IV	21(63.64%)	12(36.36%)	
Tumor size (cm)			0.0510
<5	17(40.48%)	25(59.52%)	
≥5	18(64.29%)	10(35.71%)	
Histological differentiation			0.3291
Well	16(57.14%)	12(42.86%)	
Moderate/Poor	19(45.24%)	23(54.76%)	
Lymphovascular invasion			0.0147*
Positive	19(67.86%)	9(32.14%)	
Negative	16(38.10%)	26 (61.90%)	

### ZNF674-AS1 regulated oxaliplatin resistance of gastric cancer cells

**Figure 2:**
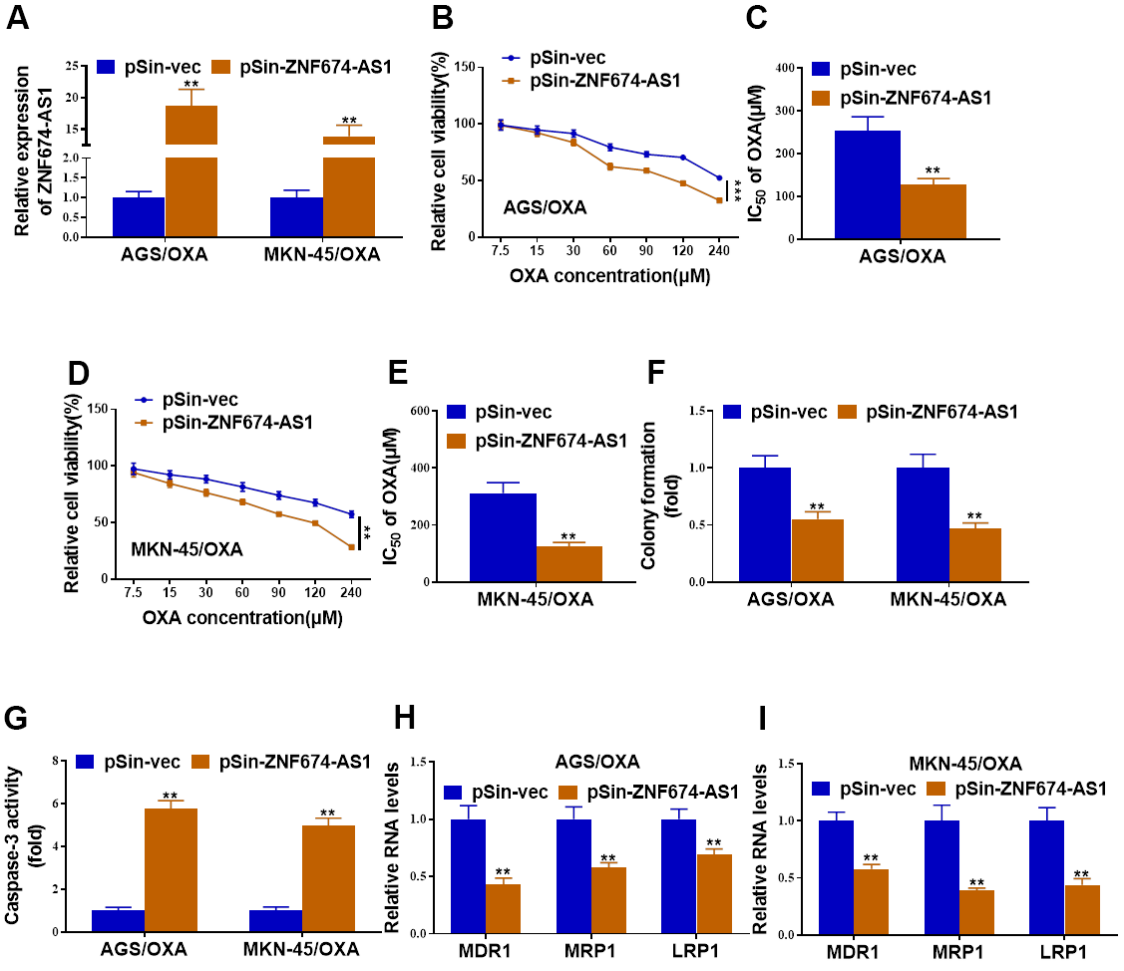
**Forced expression of ZNF674-AS1 inhibited oxaliplatin resistance of gastric cancer cells.** AGS/OXA and MKN-45/OXA cells were stably transfected with ZNF674-AS1 overexpression plasmid (pSin-ZNF674-AS1) or empty vector (pSin-vec) (**A**) The overexpression efficiency of ZNF674-AS1 was validated by qRT-PCR. (**B**–**E**) Cell viabilities were examined by CCK-8 assay and the IC_50_ value to oxaliplatin was calculated. (**F**) Clone formation of ZNF674-AS1-overexpressing or negative control AGS/OXA and MKN-45/OXA cells with the treatment of 15μM oxaliplatin. (**G**) Cell apoptosis was determined by caspase-3 activity assay, with the treatment of 15μM oxaliplatin. (**H**, **I**) mRNA levels of MDR1, MRP1 and LRP1 were evaluated by qRT-PCR. Two-way ANOVA for (**B**, **D**) Student’s t-test for others.

Next, OXA-resistant AGS and MKN-45 cells were stably transfected with ZNF674-AS1 overexpression plasmid or the empty vector (Figure 2A), ectopic ZNF674-AS1 obviously inhibited cell viability and IC_50_ value of OXA of AGS/OXA and MKN-45/OXA cells (Figure 2B–2E). Reduced colony formation capacity was observed in ZNF674-AS1-overexpressing AGS/OXA and MKN-45/OXA cells (Figure 2F). Caspase-3 activity assay indicated that forced expression of ZNF674-AS1 increased apoptosis of AGS/OXA and MKN-45/OXA cells (Figure 2G). Further, forced expression of ZNF674-AS1 deceased expression of drug resistance-related genes (MDR1, MRP1 and LRP1) (Figure 2H, 2I).

**Figure 3:**
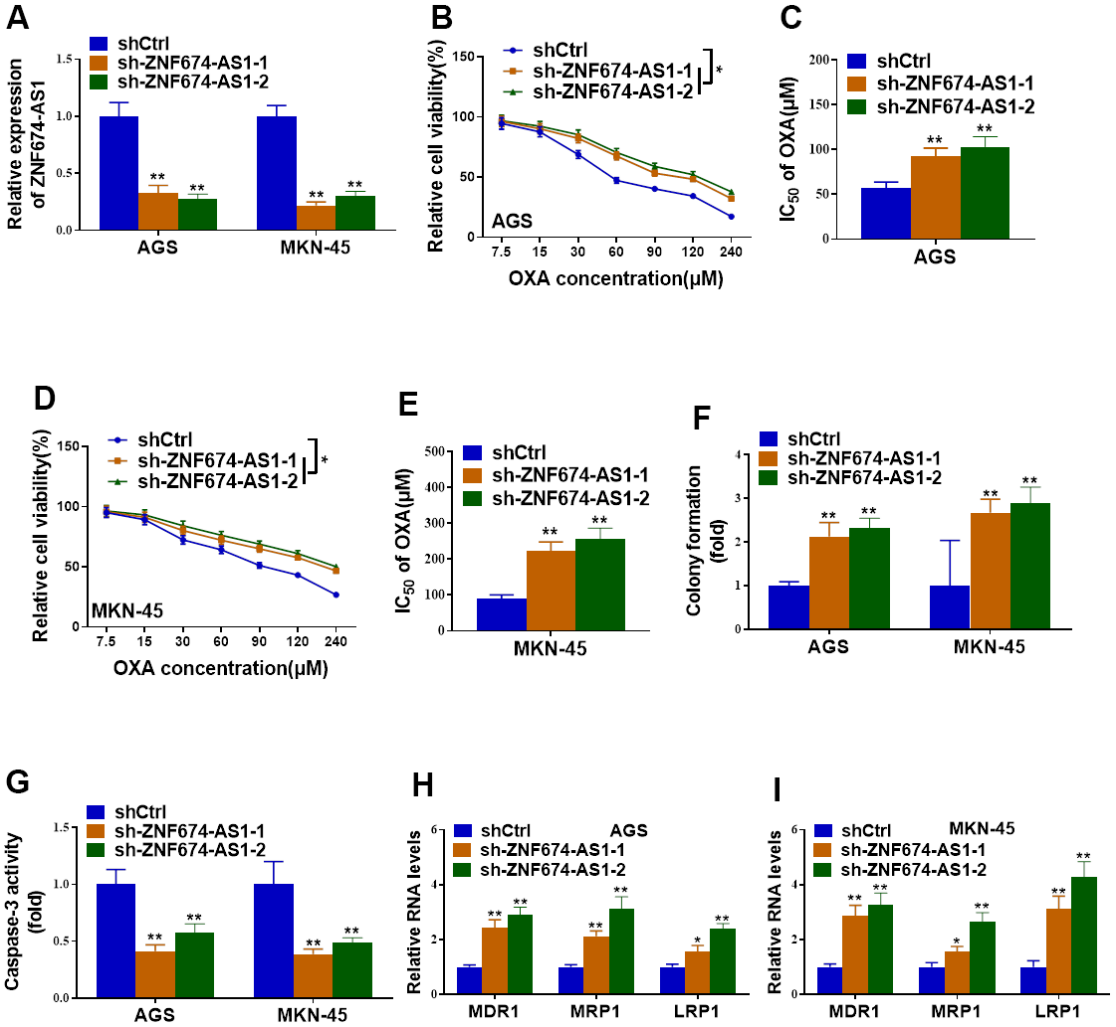
**Depletion of ZNF674-AS1 enhanced oxaliplatin resistance of gastric cancer cells.** AGS and MKN-45 cells were stably transfected with specific shRNAs against ZNF674-AS1 (sh-ZNF674-AS1-1 and sh-ZNF674-AS1-2) or empty plasmid (shCtrl) (**A**) Knockdown efficiency of ZNF674-AS1 was validated by qRT-PCR. (**B**–**E**) Cell viabilities were examined by CCK-8 assay and the IC_50_ value to oxaliplatin was calculated. (**F**) Clone formation of ZNF674-AS1-silenced or negative control AGS and MKN-45 cells, with the treatment of 5μM oxaliplatin. (**G**) Cell apoptosis was determined by caspase-3 activity assay, with the treatment of 5μM oxaliplatin. (**H**, **I**) mRNA levels of MDR1, MRP1 and LRP1 were evaluated by qRT-PCR. Two-way ANOVA for (**B**, **D**) Student’s t-test for others.

On the other hand, endogenic ZNF674-AS1 was knockdown in parental AGS and MKN-45 cells by stably transfection of shRNAs against ZNF674-AS1 (Figure 3A). Silence of ZNF674-AS1 obviously increased cell viability, IC_50_ value and colony formation capacity of AGS and MKN-45 cells with treatment of OXA (Figure 3B–3F). Cell apoptosis was remarkably decreased in ZNF674-AS1-depleted AGS and MKN-45 cells (Figure 3G). Knockdown of ZNF674-AS1 resulted in increased expression of drug resistance-related genes (Figure 3H, 3I). Taken together, our data suggested ZNF674-AS1 obviously enhanced chemosensitivity of GC cells.

### ZNF674-AS1 negatively regulated the expression of CHST7

**Figure 4:**
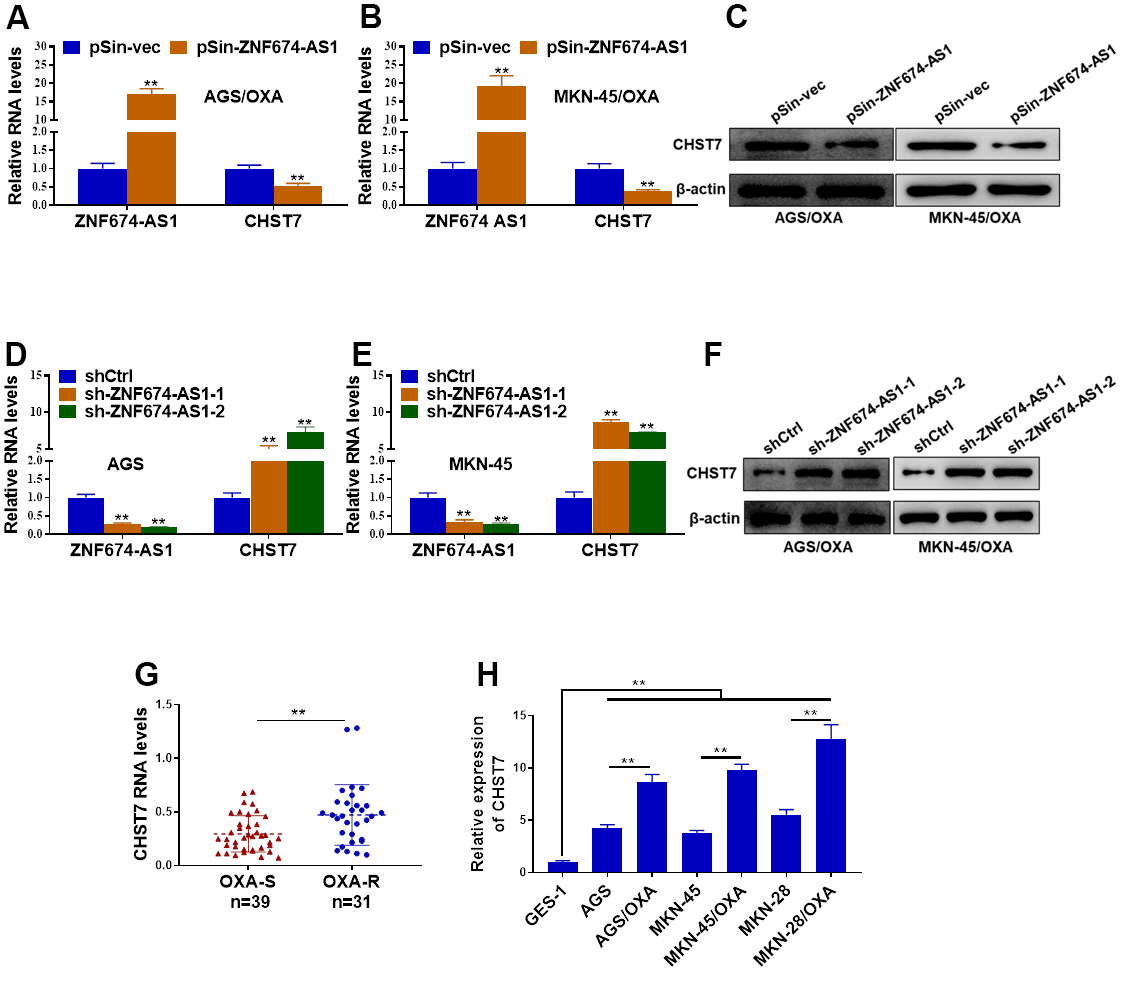
**ZNF674-AS1 exhibited suppressive effects on CHST7 expression.** (**A**, **B**) The RNA levels of ZNF674-AS1 and CHST7 in AGS and MKN-45 cells stably transfected specific shRNAs against ZNF674-AS1 (sh-ZNF674-AS1-1 and sh-ZNF674-AS1-2) or empty plasmid (shCtrl) were examined by qRT-PCR (**C**) Protein level of CHST7 in ZNF674-AS1-overexpressing or negative control AGS/OXA and MKN-45/OXA cells was determined by western blot. (**D**, **E**) The RNA levels of ZNF674-AS1 and CHST7 in AGS/OXA and MKN-45/OXA cells stably transfected ZNF674-AS1 overexpression plasmid (pSin-ZNF674-AS1) or empty vector (pSin-vec) were examined by qRT-PCR (**F**) Protein level of CHST7 in ZNF674-AS1-silenced or negative control AGS and MKN-45 cells was examined by western blot. (**G**) RNA level of CHST7 in oxaliplatin-resistant tumor tissues and oxaliplatin-sensitive tumor tissues. (**H**) qRT-PCR showed CHST7 expression in six GC cell lines (oxaliplatin-resistant cells or parental MKN-45, AGS and MKN-28) and normal human gastric epithelial cell lines. Student’s t-test.

Previous studies have reported LncRNAs mediated their neighboring genes expression via EZH2-mediated epigenetic regulation [27, 28]. To determine the molecular mechanisms underlying ZNF674-AS1-induced OVA resistance of GC cells, the neighboring CHST7 was concerned. Both protein and mRNA levels of CHST7 were decreased in AGS/OXA and MKN-45/OXA cells transfected with ectopic ZNF674-AS1 plasmid (Figures 4A–4C). In contrast, silence of ZNF674-AS1 markedly led to increased expression of CHST7 (Figure 4D–4F). More importantly, CHST7 mRNA level was notably elevated in OXA-resistant GC tissues, as compared with the OXA-sensitive counterparts (Figure 4G). Furthermore, CHST7 level was remarkably increased in six GC cell lines (AGS, AGS/OXA, MKN-45, MKN-45/OXA, MKN-28, MKN-28/OXA cells) as compared with GES-1. In addition, A higher level of CHST7 was observed in OXA-resistant GC cells than that in their parental GC cells (Figure 4H). Summarily, we proved that ZNF674-AS1 suppressed the expression of CHST7.

### ZNF674-AS1 promoted methylation of CHST7 by recruiting EZH2

**Figure 5:**
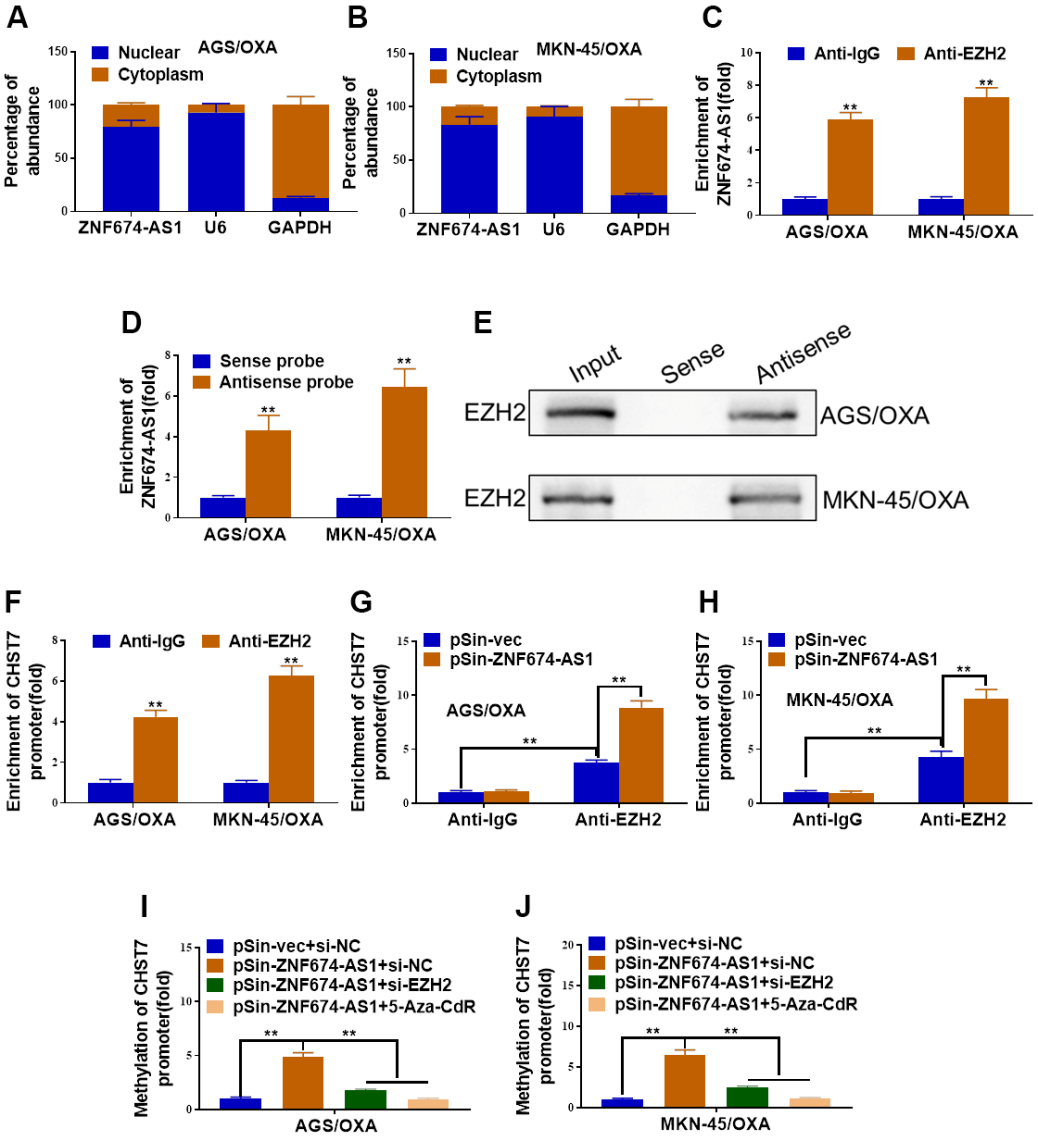
**ZNF674-AS1 promoted methylation of CHST7 by recruiting EZH2.** (**A**, **B**) qRT-PCR determined the distribution of ZNF674-AS1 in AGS/OXA and MKN-45/OXA cells. (**C**) The relative enrichment of ZNF674-AS1 immunoprecipitated by anti-EZH2 or anti-IgG in AGS/OXA and MKN-45/OXA cells was determined by RIP assay. (**D**, **E**) Biotin pull-down assay was performed to verify interaction between ZNF674-AS1 and EZH2, followed by qRT-PCR analysis to evaluate ZNF674-AS1 levels (**D**) and western blot analysis to examine EZH2 enrichment (**E**). (**F**) The relative enrichment of CHST7 promoter immunoprecipitated by anti-EZH2 or anti-IgG in AGS/OXA and MKN-45/OXA cells was assessed by ChIP-PCR assay. (**G**, **H**) The relative enrichment of CHST7 promoter immunoprecipitated by anti-EZH2 or anti-IgG in ZNF674-AS1-overexpressing or negative control AGS/OXA and MKN-45/OXA cells was evaluated by ChIP-PCR assay. (**I**, **J**) BSP analysis of the methylation levels of CHST7 promoter in AGS/OXA and MKN-45/OXA cells treated with pSin-vec+siNC, pSin-ZNF674-AS1+siNC, pSin-ZNF674-AS1+siEZH2 or pSin-ZNF674-AS1+ 5-Aza-CdR. Student’s t-test.

We further explored the specific regulatory mechanism of ZNF674-AS1-induced CHST7 inhibition. ZNF674-AS1 was verified to be primarily located in nucleus, which implied its role in gene regulation transcriptionally (Figure 5A, 5B). RIP assay demonstrated ZNF674-AS1 transcripts were observably enriched by anti-EZH2 antibody in both AGS/OXA and MKN-45/OXA cells (Figure 5C). RNA pulldown assay indicated ZNF674-AS1 directly bonded to EZH2 (Figure 5D, 5E). Meanwhile, the enrichment of EZH2 at CHST7 promoter was verified by ChIP assay (Figure 5F). Consistently, forced expression of ZNF674-AS1 promoted the recruitment of EZH2 towards CHST7 promoter (Figure 5G, 5H). Moreover, ZNF674-AS1 overexpression resulting enhanced methylation of CHST7 promoter was impaired by EZH2 silence or 5-Aza-CdR treatment (Figure 5I, 5J). Taken together, our data suggested ZNF674-AS1 epigenetically inhibited its neighboring CHST7 expression through complexation with EZH2.

### Silence of CHST7 resensitized oxaliplatin-resistant gastric cancer cells to oxaliplatin

**Figure 6:**
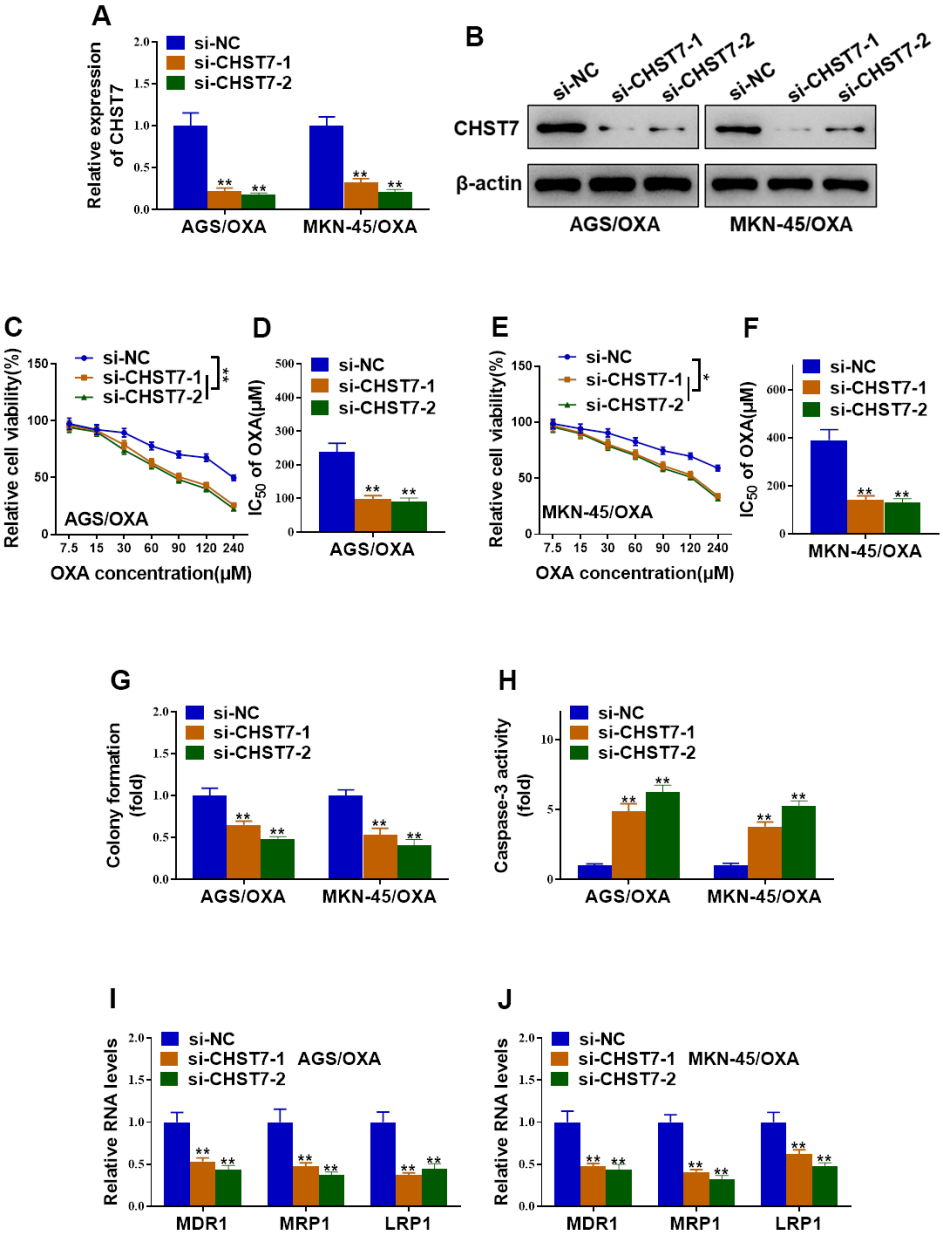
**Silence of CHST7 resensitized OVA-resistant gastric cancer cells to oxaliplatin.** (**A**, **B**) The knockdown efficiency of CHST7 in AGS/OXA and MKN-45/OXA cells transfected with CHST7 siRNAs (si-CHST7-1 and si-CHST7-2) or si-NC was validated by qRT-PCR and western blot. (**C**–**F**) Cell viability and the IC_50_ of oxaliplatin in AGS/OXA and MKN-45/OXA cells transfected with CHST7 siRNAs or si-NC were determined by CCK-8 assay. (**G**) Colony formation assay showed knockdown of CHST7 decreased cell survival of AGS/OXA and MKN-45/OXA cells exposed to 15μM oxaliplatin. (**H**) Caspase-3 activity assay indicated that knockdown of CHST7 increased apoptosis of AGS/OXA and MKN-45/OXA cells exposed to 15μM oxaliplatin. (**I**, **J**) qRT-PCR indicated that silence of CHST7 decreased expression levels of drug-resistant genes (MDR1, MRP1, LRP1) in AGS/OXA and MKN-45/OXA cells. Two-way ANOVA for (**C**, **E**) Student’s t-test for others.

To clarify whether CHST7 was involved in oxaliplatin-resistant in GC cells, specific siRNAs against CHST7 were transfected into AGS/OXA and MKN-45/OXA cells. Knockdown efficiency of CHST7 was validated by qRT-PCR and western blot analysis, respectively (Figure 6A, 6B). In contrast to ZNF674-AS1, CHST7 depletion significantly suppressed cell viability and IC_50_ value of OXA-resistant cells exposed to oxaliplatin (Figure 6C–6F). Besides, CHST7-depleted OXA-resistant cells exhibited a reduced colony formation capacity (Figure 6G). Caspase-3 activation was greatly stimulated by the knockdown of CHST7 (Figure 6H). At the molecular levels, silence of CHST7 significantly down-regulated drug-resistant genes (MDR1, MRP1, LRP1) in both AGS/OXA and MKN-45/OXA cells (Figure 6I, 6J). Therefore, our results supported that knockdown of CHST7 resensitized OXA-resistant GC cells to oxaliplatin.

## DISCUSSION

Gastric cancer is an aggressive malignant tumor accompanied by high mortality and poor prognosis [29]. Chemotherapy has been employed in GC patients to reduce the risk of metastasis and postoperative recurrence [30]. However, acquisition of drug resistance seriously impairs the therapeutic effect of chemotherapy in GC. In the present study, we constructed OVA-resistant GC cells and explored the functions and mechanisms of ZNF674-AS1 in oxaliplatin resistance of GC.

Increasing evidence suggested that LncRNAs participated in several biological processes of multiple cancer types including proliferation, epithelial-mesenchymal transition (EMT), drug resistance and metastasis [31, 32]. Despite it has been previously reported to serve as a tumor suppressor in hepatocellular and glioma cancers [17, 18], the detailed expression, functions and regulatory mechanisms of ZNF674-AS1 in GC remains to be explored. In the current work, a notably lower level of ZNF674-AS1 was observed in OVA-resistant GC samples and cells, which implied ZNF674-AS1 may participate in the mediation of oxaliplatin resistance in GC.

EZH2 inhibits expression of target genes by catalyzing the formation of H3K27me3 [33]. More and more evidence has suggested that LncRNAs acted as a crucial role in EZH2-mediated epigenetic regulation in multiple cancer types. For example, elevated LINC01088-induced proliferation advantage depended on EZH2-mediated epigenetically inhibition of p21 expression in human non-small cell lung cancer [34]. Another study suggested that LINC01535 enhanced distant metastasis via mediating EZH2-targeted miR-214 in cervical cancer [35]. In addition, a recent study has reported that STXBP5-AS1 inhibited cancer stem cell phenotypes of pancreatic cancer cells through EZH2/ADGB pathway [26]. Here, we demonstrated ZNF674-AS1 directly bond to EZH2, led to expression inhibition of its neighboring CHST7 epigenetically, resulting in impairment of oxaliplatin resistance in GC. However, how does ZNF674-AS1 recruit EZH2, it is still needed to further determine other factors involved.

Carbohydrate Sulfotransferase 7 (CHST7) belongs to the CHST family, which undertakes the function to transfer sulfate to carbohydrate groups in glycoproteins [36]. Fewer studies has reported CHST7 was associated with carcinogenic process. A previous literature uncovered that serum CHST7 was significantly increased in all overall stages of primary lung tumors [37]. In addition, Bi et al. reported that methylation level of CHST7 in white blood cells was correlated to colorectal cancer risk [38]. In the current study, we firstly revealed the expression pattern and functions of CHST7 in oxaliplatin resistance of gastric cancer.

## CONCLUSIONS

**Figure 7:**
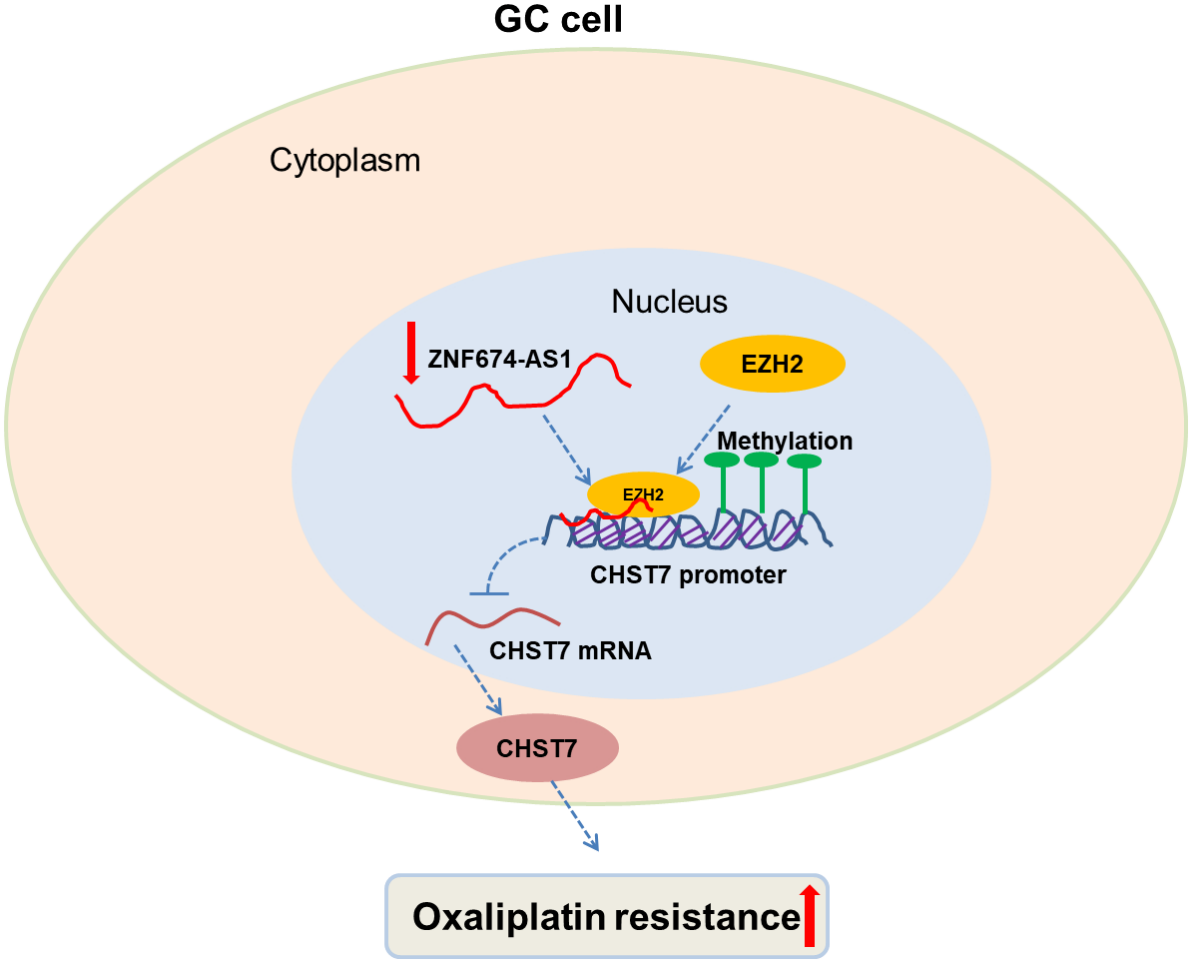
**Proposed model for the tumor suppressive role of ZNF674-AS1 in oxaliplatin resistance of GC.** ZNF674-AS1 epigenetically silences CHST7 expression via binding to EZH2, which inhibits oxaliplatin resistance of GC.

In our work, we demonstrated that ZNF674-AS1 and CHST7 exhibited two opposite expression patterns and functions during regulation of oxaliplatin resistance in GC, which was due to ZNF674-AS1-induced inhibition of CHST7 at transcriptional level (Figure 7). Our study potentially provided novel therapeutic targets and strategies for GC patients suffering from oxaliplatin resistance.
